# Key Features of Intertidal Food Webs That Support Migratory Shorebirds

**DOI:** 10.1371/journal.pone.0076739

**Published:** 2013-10-25

**Authors:** Blanche Saint-Béat, Christine Dupuy, Pierrick Bocher, Julien Chalumeau, Margot De Crignis, Camille Fontaine, Katell Guizien, Johann Lavaud, Sébastien Lefebvre, Hélène Montanié, Jean-Luc Mouget, Francis Orvain, Pierre-Yves Pascal, Gwenaël Quaintenne, Gilles Radenac, Pierre Richard, Frédéric Robin, Alain F. Vézina, Nathalie Niquil

**Affiliations:** 1 Université de la Rochelle-CNRS, UMR 7266, Littoral Environnement et Sociétés (LIENSs), La Rochelle, France; 2 Laboratoire d’Océanographie Biologique de Banyuls, Banyuls, France; 3 Université Lille 1, Laboratoire d’Océanographie et de Géoscience, Wimereux, France; 4 Université du Maine, Ecophysiologie et Métabolisme des Microalgues, Le Mans, France; 5 UFR Sciences Exactes et Naturelles (SEN), Département de Biologie, Pointe à Pitre, France; 6 Bedford Oceanographic Institute, Dartmouth, Canada; 7 CNRS, UMR 7208, Biologie des Organismes et Ecosystèmes Aquatiques (BOREA), Laboratoire Biologie des Mollusques Marins et des Ecosystèmes Associés (BioMea), Caen, France; University of Western Ontario, Canada

## Abstract

The migratory shorebirds of the East Atlantic flyway land in huge numbers during a migratory stopover or wintering on the French Atlantic coast. The Brouage bare mudflat (Marennes-Oléron Bay, NE Atlantic) is one of the major stopover sites in France. The particular structure and function of a food web affects the efficiency of carbon transfer. The structure and functioning of the Brouage food web is crucial for the conservation of species landing within this area because it provides sufficient food, which allows shorebirds to reach the north of Europe where they nest. The aim of this study was to describe and understand which food web characteristics support nutritional needs of birds. Two food-web models were constructed, based on *in situ* measurements that were made in February 2008 (the presence of birds) and July 2008 (absence of birds). To complete the models, allometric relationships and additional data from the literature were used. The missing flow values of the food web models were estimated by Monte Carlo Markov Chain – Linear Inverse Modelling. The flow solutions obtained were used to calculate the ecological network analysis indices, which estimate the emergent properties of the functioning of a food-web.

The total activities of the Brouage ecosystem in February and July are significantly different. The specialisation of the trophic links within the ecosystem does not appear to differ between the two models. In spite of a large export of carbon from the primary producer and detritus in winter, the higher recycling leads to a similar retention of carbon for the two seasons. It can be concluded that in February, the higher activity of the ecosystem coupled with a higher cycling and a mean internal organization, ensure the sufficient feeding of the migratory shorebirds.

## Introduction

The French Atlantic coast constitutes one of the southernmost attractive areas for shorebird populations wintering in Europe. This is the case of the Pertuis Charentais, where birds use the network of estuarine bays as a stopover or wintering area along the East Atlantic flyway. The Pertuis Charentais is composed of various habitats, largely dominated by intertidal mudflats, which are among the largest in Europe [1]. One of the largest and most-studied sites in the Pertuis Charentais is the eastern mudflat (Brouage mudflat) inside the Marennes-Oléron Bay, which is important for oyster and mussel farming. The central part of the mudflat and adjacent marshlands are included in the National Nature Reserve of Moëze-Oléron (about 6,700 hectares).

Every year, c. 66,000 shorebirds use the Marennes-Oléron Bay in mid-winter, with most of them foraging within the limits of the nature reserve. Among the 18 species present in the bay, six are common and represent c. 85% of all individuals in winter (Red Knot, *Calidris canutus*; Dunlin, *Calidris alpina*; Black-tailed Godwit, *Limosa limosa*; Common Redshank, *Tringa totatnus*; Grey Plover, *Pluvialis squatarola*; and Curlew, *Numenius arquata*) [2]. The Shelduck, *Tadorna tadorna* is the only anatidae common on bare mudflats. The intertidal mudflat serves one of two functions for shorebirds, depending on the migrating schedules of the considered populations. Shorebirds might use the area for winter survival and stay in the region for most of the non-breeding season or they might use it for refuelling during stopover when migrating further south. The Marennes-Oléron Bay appears to be one of the most attractive sites for coastal shorebirds in the Pertuis Charentais, due to the easy access to huge bare mudflats used as a foraging area and to the presence of a high tide roost in nearby marshland. Moreover, the nature reserve is a classified protected area where hunting is strictly forbidden [3]. Most of the shorebird species wintering in the bay breed in northern Europe, Siberia, Greenland or the Canadian Arctic [4]. Consequently, birds are present in the bay from August to May, with a peak number around January. During the winter, birds feed to fulfil their daily energy needs for survival and to refuel at the end of winter before flying towards their breeding areas. They feed on the tidal Brouage mudflat on macrofauna species, particularly molluscs and annelids. For instance, the gastropods *Hydrobia ulvae* (new name: *Peringia ulvae*) contribute to about 85% of the diet of the Common Shelduck (*T tadorna*) [5] and the Red Knot [6].

The Brouage mudflat is a bare mudflat (i.e. no seagrass or macroalgae grows on this site) and its primary production is mainly due to the microphytobenthos composed of benthic diatoms [7]. At low tide, diatoms and associated bacteria are concentrated in the first few centimeters of the sediment [8] and form a biofilm that supports the benthic food web. Meiofauna and deposit feeders (e.g. *Hydrobia*), comprising herbivorous and bacterivorous species, feed on the biofilm. In summer, there is less predation on the benthic macrofauna and their biomass accumulates. In winter, birds feed on macrofaunal species.

The aim of this study was to understand which ecosystem characteristics support the wintering of the birds. To do this, the structure and functioning of this ecosystem were modelled in two seasons; one with a large number of shorebirds (winter) and the other one when shorebirds were absent (summer). This comparative study aims to highlight which features of the ecosystem are crucial in sustaining such a high predator biomass. In regard to the previous result models on the Brouage mudflat, the lower primary and secondary productions observed during winter [9,10] suggested two main features for the winter food web: 1) a higher efficiency in the transfer of carbon via a higher specialization of trophic links, 2) a stronger cycling in order to increase the stock of carbon available for the shorebirds and thus sustain their nutritional needs.

The ecosystem flows that were not estimated *in situ* during field campaigns, were estimated using Monte Carlo Markov Chain – Linear Inverse Modelling (MCMC-LIM) [11,12,13]. The set of possible solutions for each flow of the benthic food web in winter and summer, resulting from the MCMC-LIM method, was used to calculate indices of ecological network analysis (ENA). The ENA indices are used to characterise the overall structural properties of food webs, including activity, recycling, specialisation, trophic efficiency, and mean path length [e.g. 14]. ENA indices constitute a set of indices that describe the connections between compartments through an analysis of the input and output flows of a compartment, the trophic structure based on a linearisation of the network and the degree of redundancy or specialisation of the flows [14,15,16]. The set of solutions of flows obtained by MCMC-LIM, allowed the calculation of ranges and confidence intervals for some of these indices and thus facilitated statistical tests to compare the two seasonal food webs.

## Material and Methods

### 1: Study area

**Figure 1:**
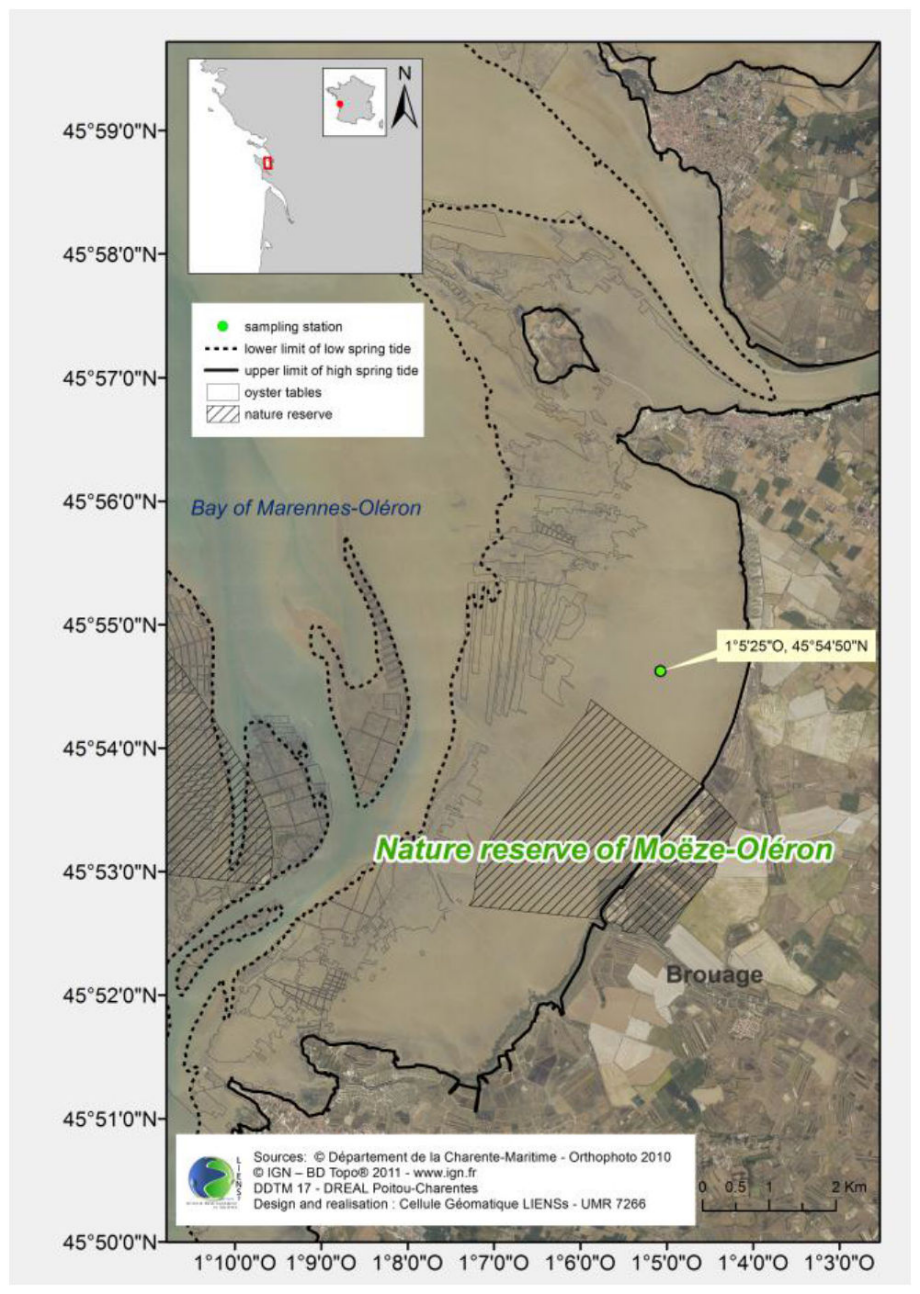
Study site the Brouage mudflat that includes a part of the nature reserve of Moëze-Oléron. Published with permission under a CC-BY license.

The Brouage intertidal mudflat is located on the French Atlantic coast in the bay of Marennes-Oléron (Figure 1). The bay covers 150 km^2^ and the Brouage mudflat, which is located in the eastern part of the bay, occupies 68 km^2^ at low tide. The bottom slope is relatively flat (1:1,000) and the tidal area is large (up to 4 km). The sediment consists of silt and clay particles (95% < 63 µm) [17]. The sampling zone for this study was located in the centre of the Brouage mudflat and is characterised by a typical ridge and runnel structure [18]. It is located at about 1.5 km from the lower part of this intertidal zone, an area covered by oysters from both abandoned and active oyster farms.

### 2: Field measurements

Two field campaigns were performed; in winter (from 16 February to 24 February 2008) and in summer (from 13 July to 26 July 2008). Measurements were taken at low tide during the neap-spring cycle. No specific permission was required for the field sampling because the sampling site was located outside the nature reserve of Moëze-Oléron. Moreover, no endangered or protected species were involved in the field sampling. The species included the categories described below.

#### 2.1: Microphytobenthos

Algal biomass in the sediment was estimated using chlorophyll *a* as a proxy, measured using fluorometry. The carbon algal biomass was estimated from the chlorophyll *a* biomass by the carbon/chlorophyll *a* ratio of 45 [19]. Irradiance at the mudflat surface (µmol photons m^-2^ s^-1^) was estimated from Meteo France records and a Licor quantometer.

The mean fluxes of gross primary production and exudation of microphytobenthos were calculated from fortnightly simulations of the coupled dynamics of microphytobenthos, bacteria and extracellular polymeric substances (EPS) under tide, light and temperature. The simulations were performed using a dynamic model adapted from Guarini et al. [20], regulating the migratory dynamics of the microphytobenthos in the sediments by nitrogen and carbon internal quotas (Guizien pers. comm.).

#### 2.2: Bacteria

To estimate bacterial abundance, bacteria were extracted from cores of sediment by dilution with sodium pyrophosphate (0.01 mol L^-1^ for >30 min at 4°C). Bacteria were stained with DAPI (2.5 mg L-1) for 15 min in darkness, filtered through 0.2 µm Nucleopore black filters and counted with an epifluorescence microscope (x1,000, Axioskop 2 mot plus, Zeiss). The bacterial biovolume (V) was estimated from the length and the width using Fuhrman’s formula [21]. The carbon contained in a bacterium was calculated based on the formula 133.754*V^0.438^(Vin µm^3^) [22] and was estimated as equal to 79 fg C. cell^-1^ for a mean biovolume of 0.28 µm^3^. This estimate was used to determine the biomass of bacteria in carbon equivalents. The production of sediment-inhabiting bacteria was estimated from their biomass and the ratio of production/biomass, which was previously determined in 2006 for February and July [17].

The *in situ* viral production was estimated as the change in viral abundance after 3 h divided by the time elapsed, for three replicates. The bacterial mortality induced by the viral lysis was determined from the viral production divided by a burst-size of 36 [23], corresponding to the number of virus particles produced per bacterium.

#### 2.3: Meiofauna

The abundance of the meiofauna (foraminifera, copepods and nematodes) in the sediment was estimated at the slack water tide; three replicates were performed each time. The meiofauna was extracted using Ludox HS40 and was counted using a Motoda-box to split samples and obtain aliquots with a number of individuals exceeding 500. The biomass of the meiofauna and its bacterivory were taken from a previous seasonal study at the Brouage mudflat [17]. The bacterivory was measured with *in situ* experiments based on ^15^N-enriched bacteria [24]. The grazing rate of the microphytobenthos by the nematodes was previously estimated using a culture of diatoms, *Navicula jeffreyi*, pre-labeled with ^14^C [25].

#### 2.4: Macrofauna

The abundance and biomass of the macrofauna were estimated by randomly choosing three quadrants of 4 m^2^ for sampling, using three sediment cores (30.5 cm in diameter). The resulting nine replicates were sieved over a 1 mm mesh and preserved in ethanol 70%. Determination to the species level and counting were performed following rose Bengal staining under a binocular microsope. The samples were then dried in an incubator (50°C) for 24 h and incinerated at 540°C, to estimate the biomass as ash-free dry mass. The comparison of the isotope signatures (δ ^13^C, δ ^15^N) of sources (microphytobenthos, the benthic and pelagic detrital organic matter) with those of the macrofauna allows the determination of the contribution of each resource to the diet of macrofauna. Samples were analysed using an elemental analyser (Flash EA 1112, Thermo Scientific, Milan, Italy), coupled to an isotope ratio mass spectrometer (Delta V Advantage with a Conflo IV interface, Thermo Scientific, Bremen, Germany). Results are expressed in the δ unit notation as deviations from standards (Vienna Pee Dee Belemnite for δ^13^C and N_2_ in air for δ^15^ N), following the formula: δ^13^C or δ^15^N = ((*R*_sample/_ *R*_standard_)-1) x 10^3^, where *R* is^13^C/^12^C or ^15^N/^14^N. Reference gas calibration was performed using reference materials (USGS-24, IAEA-CH6, IAEA-600 for carbon; IAEA-N1, -N2, -N3, -600 for nitrogen). Analytical precision based on isotope values of acetanilide (Thermo Scientific) was used to estimate the C and N content for each sample series and was <0.1% both for carbon and nitrogen.

#### 2.5: Shorebirds

Shorebird abundance in the Marenne–Oléron Bay was established by counts in January 2008, according to the International Wetland Census [2]. Further studies on the diet and the time budget, defined as the time birds spent feeding, were performed for the Black-tailed Godwit, the Red Knot, the Curlew and the Shelduck. The diet of the birds was determined from dropping analysis following the methods in Dekinga and Piersma [26] or Scheiffarth [27] ).

Recent studies have shown that some shorebird species, especially the Dunlin and the Western Sandpipers, can graze on microphytobenthos [28,29]. However, no trophic link between the microphytobenthos and the shorebird species present on the Brouage mudflat during winter could be demonstrated by isotopic analysis (Bocher, pers. comm.). For that reason, grazing of the microphytobenthos by shorebirds was not considered in the food web models in this study.

### 3: Food-web construction

Two food webs were constructed, either with shorebirds (in winter) or without (in summer). Compared to previous models published for the Brouage mudflat [10,30,31], the current models consider shorter time spans and spatial scales. The food webs represent the trophic interactions between species during a mean daily low tide in February and July in the mid-zone of the mudflat. The unknown flows were reconstructed based on the Monte Carlo Markov Chain – Linear Inverse Modeling (MCMC-LIM) method. Four successive steps were necessary for the construction of the food web models [13].

#### 3.1: Topology of the food web

The first step determined all the compartments and the flows linking the species. The summer food web was composed of 12 compartments (Table 1). The seven bird species present on the mudflat during winter were combined into a single so that the winter food web contained a total of 13 compartments (Table 1). Fourteen macrofauna species were assembled into five groups (deposit feeder, carnivorous, omnivorous, suspension feeder and facultative suspension feeder), according to their diet and their trophic behaviour [32,33].

**Table 1:** Compartments composing the winter and summer food webs.

**Compartments**	**Abbreviations**
Microphytobenthos	mpb
Benthic bacteria	bcb
Foraminifera and copepods	mfb
Nematodes	nem
Deposit feeders (mainly Hydrobia ulvae*)	dep
Suspension feeders	sus
(mainly Cerastoderma edule and Ruditapes philippinarum**)	
Facultative suspension feeders (*Macoma balthica*)	suf
Omnivorous species (mainly H*ediste diversicolor*)	omn
Carnivorous species (mainly *Nephtys hombergii*)	car
Carnivorous birds	cbr
Benthic viruses	vrb
Benthic particulate carbon	bpc
Benthic dissolved carbon	bdc

* New name: *Peringia ulvae* ** new name: *Venerupis philippinarum*

The natural mortality of microphytobenthos and benthic bacteria was considered as negligible, based on the importance of the grazing observed in the field. Only the EPS (Extracellular polymeric substance) exudation of the microphytobenthos to the dissolved organic carbon (bdc) via secretion was considered. A flow of dissolved organic carbon exudation by bacteria was included; part of this flow corresponds to the loss of bacterial carbon via viral lysis. Part of the carbon originating from bacteria was considered as available for consumers during the high tide. The consumption by the five macrofauna groups was considered as equal over the 24 h diurnal cycle, irrespective of the tidal level, even for the deposit feeders (mainly composed of *H. ulvae* [34]). As consequences, links corresponding to unconsumed production between high and low tides were considered as imports. The summer and winter food web models were composed of 62 and 70 flows, respectively.

#### 3.2: Equations

The second step consisted of establishing a set of linear equations (Table 2). The mass balances that correspond to the sum of the inflows and outflows for each of the compartments constitute the first part of the linear equations. The variations in the compartment biomasses are generally considered negligible, compared to the flow values, which provide mass balances equal to zero. The second part of the linear equations was composed of the flows that were locally estimated in the sampling area. The resulting set of linear equations was written in the form , where is the vector that contains the possible flows, is the matrix that expresses the mass balance and the field observation as a combination of coefficients of the carbon flows, and is the vector that contains the values of mass balances and the values of the known flows [35].

**Table 2:** Mass balances (1-13) and values of flows measured in the field (14-20).

**Mass balances**		
1-Microphytobenthos	(gppTOmpb)-(mpbTOres+mpbTObdc+mpbTOmfb+mbpTOnem+mpbTOdep+mpbTOomn+mpbTOsuf+mpbTOexp)=0	
2-Benthic bacteria	(bdcTObcb)-(bcbTOres+bcbTObdc+bcbTOmfb+bcbTOnem+bcbTOdep+bcbTOomn+bcbTOsuf+bcbTOvrb)=0	
3-Foraminifera	(mpbTOmfb+bcbTOmfb+bpcTOmfb)-(mfbTOres+mfbTOcar+mfbTOomn+mfbTOcbr+mfbTObpc)=0	
4-Nematodes	(mpbTOnem+bcbTOnem+bpcTOnem)-(nemTOres+nemTOcar+nemTOomn+nemTObpc)=0	
5-Carnivorous	(impTOcar+mfbTOcar+nemTOcar+depTOcar+susTOcar+sufTOcar)-(carTOres+carTObpc+carTOcbr+carTOexp)=0	
6-Deposit feeders	(impTOdep+mpbTOdep+bcbTOdep+bpcTOdep)-depTOres+depTOcar+depTOomn+depTOcbr+depTObpc+depTOexp)=0	
7-Omnivorous	(impTOomn+mfbTOomn+nemTOomn+depTOomn+susTOomn+sufTOomn+bcbTOomn+bcpTOomn)-	
	(omnTOres+omnTOcbr+omnTObpc+omnTOexp)=0	
8-Suspension feeders	(impTOsus)-(susTOres+susTOcar+susTOomn+susTOcbr+susTObpc+susTOexp)=0	
9-Facultative suspension feeders	(impTOsuf+mpbTOsuf+bcbTOsuf+bpcTOsuf)-(sufTOres+sufTOcar+sufTOomn+sufTOcbr+sufTObpc+sufTOexp)=0	
10-Carnivorous birds	(mfbTOcbr+carTOcbr+depTOcbr+omnTOcbrsusTOcbr+sufTOcbr)-(cbrTOres+cbrTObpc+cbrTOexp)=0	
11-Benthic viruses	(bcbTOvrb)-(vrbTObdc+vrbTOext)=0	
12-Benthic particular carbon	(mfbTObpc+nemTObpc+carTObpc+depTObpc+omnTObpc+susTObpc+sufTObpc+**cbrTObpc**)-	
	(bcpTOmfb+bcpTOnem+bpcTOdep+bpcTOomn+bpcTOsuf+bpcTOexp)=0	
13-Benthic dissolve carbon	(mpbTObdc+bcbTObdc+bpcTObdc)-(bdcTObcb+bdcTOexp)=0	
**Processes\ Season**	**Winter**	**Summer**
14-gppTOmpb	413.09	183.6
15-mpbTObdc	110.75	51
16-Production of bcb (bdcTObcb-bcbTOres)	169.32	93.94
17-bcbTOmfb	0.086	0.035
18-bcbTOnem	0.107	0.11
19-bcbTOdep	6.11	11.25
20- bcbTOvrb	1.88	3.58

Flows that were only present in the winter model are in bold. The values of flows were expressed in mgC.m^- 2^ per low tide. The flows were coded by 8 letters (e.g. mpbTOmfb): the three first letters correspond to the compartment ‘source’, letters after the TO described the compartment ‘sink’ (see table 1 for compartment abbreviations). For instance the flow mpbTOmfb is the flow of carbon that leaves the compartment mpb to come into the compartment mfb. gpp= gross Primary Production, res=respiration, imp=imports and exp= exports.

#### 3.3: Inequalities

The third step consisted of adding constraints determined from the literature, experiments, or field measurements from comparable intertidal mudflats, to obtain biologically and ecologically realistic flow values. The biological constraints were expressed as a set of linear inequalities in the form: , where is the matrix that contains the coefficients of the biological constraints and is the vector which was composed of the values of these biological constraints [35].

For all the compartments, the respiration, consumption, excretion and import flows were constrained by lower and upper limits. The inequalities, which corresponded to the physiological rates (e.g. assimilation efficiency) or the diet contribution, are listed in Table 3. The other inequalities, which corresponded to the absolute values of the biological processes, are described below. Two different densities of birds were considered, to determine the maximum and minimum values of the previously cited processes (i.e. consumption, respiration and egestion). The minimum and maximum densities of shorebirds per m^2^ mudflat were based on the following hypotheses: 1) the lower density was based on the assumption that the birds covered the whole mudflat at low tide, 2) the maximum density was based on the assumption that the birds followed the ebbing tide covering only a limited area of the mudflat.

**Table 3:** Set of inequalities used (biological rates, contribution of the microphytobenthos (mpb) and of the benthic particulate carbon (bpc) to the diet of macrofauna).

Respiration	Microphytobenthos	Lower limit	0.05*gpp-resp	<0	[82]
		Upper limit	-0.3*gpp+resp	<0	
Net Growth Efficiency	Meiofauna	Lower limit	0.5*CTOmfb-0.5*mfbTOE-resp	<0	[44]
		Upper limit	-0.7*CTOmfb+0.7* mfbTOE+resp	<0	
	Nematods	Lower limit	0.1*CTOnem-0.1*nemTOE-resp	<0	[44]
		Upper limit	-0.4*CTOnem+0.4* nemTOE+resp	<0	
	Macrofauna	Lower limit	0.3*CTOmac+0.3* ITOmac-0.3*macTOE-resp	<0	[44]
		Upper limit	-0.5*CTOmac-0.5* ITOmac+0.5* macTOE+resp	<0	
	Benthic bacteria	Lower limit	0.39*U_doc_-resp	<0	[36]
		Upper limit	-0.89*U_doc_+resp	<0	
Egestion	Nematods	Lower limit	0.7*CTOnem-nemTOE	<0	[44]
		Upper limit	-0.94*CTOnem+nemTOE	<0	
	Meiofauna	Lower limit	0.23*CTOmfb-mfbTOE	<0	[44]
		Upper limit	-0.43*CTOmfb+mfbTOE	<0	
	Macrofauna	Lower limit	0.25*CTOmac-macTOE	<0	[44]
		Upper limit	-0.6*CTOmac+macTOE	<0	
Contribution of mpb to the diet of macrofauna	***In summer***				
	Deposit feeders	Upper limit	0*mpbTOdep-1*bpcTOdep	<0	in this study
	Omnivorous species	Lower limit	-0.74*mpbTOomn+0.26*bpcTOomn	<0	
		Upper limit	0.09*mpbTOomn-0.91*bpcTOomn	<0	
	Suspension feeders	Lower limit	-0.35*mpbTOsuf+0.65*bpcTOsuf	<0	
		Upper limit	0.18*mpbTOsuf-0.82*bpcTOsuf	<0	
	***In winter***				
	Deposit feeders	Lower limit	-0.5*mpbTOdep+0.5*bpcTOdep	<0	in this study
		Upper limit	-1*bpcTOdep	<0	
	Omnivorous species	Lower limit	-0.35*mpbTOomn+0.65*bpcTOomn	<0	
		Upper limit	0.14*mpbTOomn-0.86*bpcTOomn	<0	
	Suspension feeders	Lower limit	-0.24*mpbTOsuf+0.76*bpcTOsuf	<0	
		Upper limit	0.01*mpbTOsuf-0.99*bpcTOsuf	<0	
Contribution of bpc to the diet of macrofauna	***In summer***				
	Deposit feeders	Upper limit	0.89*bpcTOdep-0.11*mpbTOdep	<0	in this study
	Omnivorous species	Lower limit	-1*bpcTOomn	<0	
		Upper limit	0.56*bpcTOomn-0.44*mpbTOomn	<0	
	Suspension feeders	Lower limit	-1*bpcTOsuf	<0	
		Upper limit	0.71*bpcTOsuf-0.29*mpbTOsuf	<0	
	***In winter***				
	Deposit feeders	Lower limit	-1*bpcTOdep	<0	In this study
		Upper limit	0.69*bpcTOdep-0.31*mpbTOdep	<0	
	Omnivorous species	Lower limit	-1*bpcTOomn	<0	
		Upper limit	0.76*bpcTOomn-0.24*mpbTOomn	<0	
	Suspension feeders	Lower limit	-1*bpcTOsuf	<0	
		Upper limit	0.83*bpcTOsuf-0.17*mpbTOsuf	<0	

Gpp= gross primary production, resp=respiration, C=consumption, E=egestion. For compartments’ abbreviations see table 1.

#### 3.3.1: Respiration

The respiration of the sediment-inhabiting bacteria was constrained by the bacterial growth efficiency (BGE) between 0.11 and 0.61 as previously determined for a coastal bay [36]. The meiofauna respiration (nematodes, copepods and foraminifera) was constrained by the organic carbon biomass-specific respiration rate, which ranged between 1.6 and 2.5 µl O_2_ h^-1^ mg C^-1^. The O_2_ content was converted into carbon based on the following equality: 1 mL O_2_ = 0.4 mg C [derived from 37, in 38]. The macrofauna respiration was estimated from the range of values given by the virtual handbook of Thomas Brey (http://www.thomas-brey.de/science/virtualhandbook; Brey [39,40]), using the biomasses of the macrofauna groups and the mean temperatures derived from the field measurements (8°C and 21°C in winter and summer, respectively). In this model, the energetic expenditure of the birds was considered to be equal to the basal metabolic rate, due to the low activity of shorebirds during wintering. Shorebird respiration was estimated by an allometric relation of the basal metabolic rate (BMR) [41,42]. The respiration rate per m^2^ depends on the density of shorebirds on this area. The maximum and minimum respiration values per m^2^ mudflat were derived from the above-described limits of the shorebird densities.

#### 3.3.2: Consumption

The total consumption by nematodes was determined from the ranges of total ingestion per individual determined by Schiemer [43]. The maximum and minimum limits of macrofauna consumption were determined from its production. Production was estimated from the production/biomass (P/B) ratio, which ranged from 0.01 to 0.05 [44]. Consumption was estimated by the production/consumption ratio, with a maximum value of 0.87 [39]. The respective contributions of microphytobenthos and benthic particulate carbon to the macrofauna diet were obtained by isotope signatures. For each resource, the maximum and minimum obtained values limited their respective contribution to the macrofauna diet. The diet contribution of each trophic group was obtained by averaging the diet contribution (generated by the ISOSOURCE model), weighted by the biomass for each of the species forming a given group. In winter, shorebirds are energy minimisers and their consumption is balance by their energy expenditure [45,46]. The consumption by the shorebirds was estimated from the daily energetic requirement (C = n* 3*BMR* (1/AE), where n is the number of individuals and AE is the assimilation efficiency [41,42]). Because the maximum and minimum consumption limits for the shorebirds were derived from the above-described density limits, they were expressed per m^2^ of mudflat. Shorebirds cannot feed all day and are constrained by the time of emersion (i.e. the hourly consumption is higher than if they could or would feed the whole day). The time devoted to feeding varies according to the species (Table 4), thus, the predation pressure applied by the shorebirds per m^2^ per hour differs according to the species. Moreover, each species has a particular diet (Table 4). Knowing the contribution of the different macrofauna species to the diet of the shorebird species allowed determination of the maximum and minimum consumption of each macrofauna group by the shorebirds. The diet composition of four species (Red Knot, Black-tailed Godwit, Common Shelduck and Curlew) was determined within the study area [5,6,47,48]. The contribution of the macrofauna to the diet of these species was expressed according to the total consumption by the shorebirds and was considered as the minimum contribution of macrofauna to the total consumption by shorebirds. The diets of the three other species, which remained unknown for the study area, were not extracted from the literature due to their great variability between sites in Europe and were consequently not used to define any constraints.

**Table 4:** Time spent to feed by each shorebirds species and their diet.

**Species**	**Time budget**	**References**	**Diet**	**References**
Black-tailed Godwit (*Limosa limosa*)	10 hours	[48]	Macoma balthica: 100%	[48]
Common Redshank (*Tringa totanus*)	70% during the night, 50% during the day	[83]	Not determined for this area	-
Common Shelduck (*Tadorna tadorna*)	14 hours	[5]	Hydrobia *ulvae*: 85%	[5]
			Others: 15%	
Curlew (*Numenius arquata*)	7 hours	[84]	*Scrobicularia plana*: 22.2%	[47]
			Nephtys hombergii	
			+ Hediste *diversicolor*: 65.52%	
			Carcinus maenas: 9.9%	
Dunlin (*Calidris alpina*)	10 hours	[85]	Not determined for this area	-
Grey Plover (*Pluvialis squatarola*)	40% of the consumption during the nigth	[86]	Not determined for this area	-
Red Knot (*Calidris canutus*)	10 hours	[6]	Hydrobia *ulvae*: 86%	[6]
			Macoma balthica: 13%	
			Cerastoderma edule: 1%	

#### 3.3.3: Egestion

The egestion of the nematodes and meiofauna was constrained by the assimilation efficiency and by the net growth efficiency rates, which are defined in Table 3. For the macrofauna, a maximum egestion value was determined from the maximum value of the consumption and the minimal coefficient of the AE. The range of excretion was determined from the maximum and minimum values of consumption, considering an AE equal to 0.80 for the shorebirds [42].

#### 3.3.4: Imports

The imports for the macrofauna were considered as the production available before the diurnal low tide. In summer, this represented the macrofauna production at high tide (diurnal and nocturnal) and nocturnal low tide. In winter, only the macrofauna production generated by the previous high tide was considered as an import, because shorebirds can feed on the mudflat during the night [49]. The maximum and minimum values of the imports were estimated from the maximum and minimum values of macrofauna production obtained as described above.

#### 3.4: Calculation of the solutions

The MCMC-LIM, based on the mirror technique defined by Van Den Meersche et al. [12], calculates several solutions and allows a direct characterization of the uncertainty. This modelling technique brings the advantage of calculating a range of possible values for each flow (i.e. a probability density function) This mirror technique reflected the proposed solutions inside the walls of the solution space until one was found to respect all the defined boundaries. The walls of the solution space corresponded to the equations and the inequalities defined in the model. This application required the definition of two parameters: the jump and the number of iterations. The jump corresponds to the length between two solutions and the number of iterations is the number of solutions sampled in the solution space [12]. In this study, a jump equal to one and 500,000 iterations were chosen to obtain an optimal coverage of the solution space. All simulations were performed using MATLAB^©^ software and with an algorithm based on a translation (made by Alain Vézina and Lauriane Campo) of the R-CRAN project package LIM-Solve [12].

### 4: Ecological Network Analysis

Ecological network analysis is a set of numerical indices that describe the overall structure and function of a food web (Table 5). For the Total System Throughput (TST), Average Mutual Information (AMI), Average Path Length (APL), Internal Relative Ascendency (IRA), Finn Cycling Index (FCI), and Comprehensive Cycling Index (CCI), a MATLAB^©^ routine was written by Carole Lebreton and Markus Schartau (GKSS Research Centre, Geesthacht, Germany), to calculate the index value for every solution estimated by MCMC-LIM. The significance of the differences between the values for the two seasons was determined by the Wilcoxon test (α = 0.01). The tested hypothesis stated that the two data sets result from a continuous distribution with similar medians.

**Table 5:** List of ENA indices calculated on the set of 500,000 solutions from the MCMC-LIM implementation.

**Indices**	**Calculation**	**Definition**	**Sources**
Total System Throughput (TST)	sum of all flows	activity of the whole ecosystem	[80]
Average Path Length (APL)	(TST-sum of imports)/ sum of imports	the average number of compartments that an atom	[15,87]
		of carbon passes through between its entry into the system and its exit	
		the system and its exit	
Finn Cycling Index (FCI)	T_c_/ TST	proportion of cycled flow in a system	[88]
Average Mutual Information (AMI)	-	degree of specialisation of flows in the network	[89]
RelativeAscendency	A/dC	fraction of the network that is organized	[80]
Internal reltive Ascendency	A_i_/DC_i_	fraction of the internal exchanges that is organized	
Overheads (O)*	dC-A	fraction of the network that is not yet organized	
Relative redundancy	R/DC	proportion of the redundancy in the network	
System trophic efficiency	logarithmic mean of the all level efficiencies	globale efficiency of transfer through the network	[32]

T_c_: quantity of carbon that is involved in cycling. A: the Ascendency (=TST*AMI). DC: Development Capacity corresponds to the maximal value of Ascendency. Ai: internal Ascendency. DC_i_: Internal development capacity. A_i_ and DC_i_ only consider internal exchanges, and thus respiration exports and imports are excluded. * Overheads are divided into three groups: 1-Overheads on the imports, 2- Overheads on the exports and 3- Dissipative overheads. All of them were expressed in percentage of the DC.

For the Lindeman spine, the relative redundancy, relative overheads, number of cycles and system trophic efficiency, the software EcoNetwrk, developed by Ulanowicz and Kay [50], was used with a unique set of solutions. This solution set corresponded for each flow, to the mean value of the whole set of possible solutions [51].

The trophic analysis, based on the trophic concept of Lindeman [52], corresponded to a representation of the complex network by a concatenated trophic chain with discrete trophic levels [32]. The quantity of transferred carbon from one level to another was represented, as well as the carbon loss by respiration, exports and egestion/excretion. The Lindeman spine allowed the calculation of the transfer efficiency from one level to the next.

## Results

### 1: Throughputs and internal flows

Details on flow values were available in the table S1 in the supporting information

**Figure 2:**
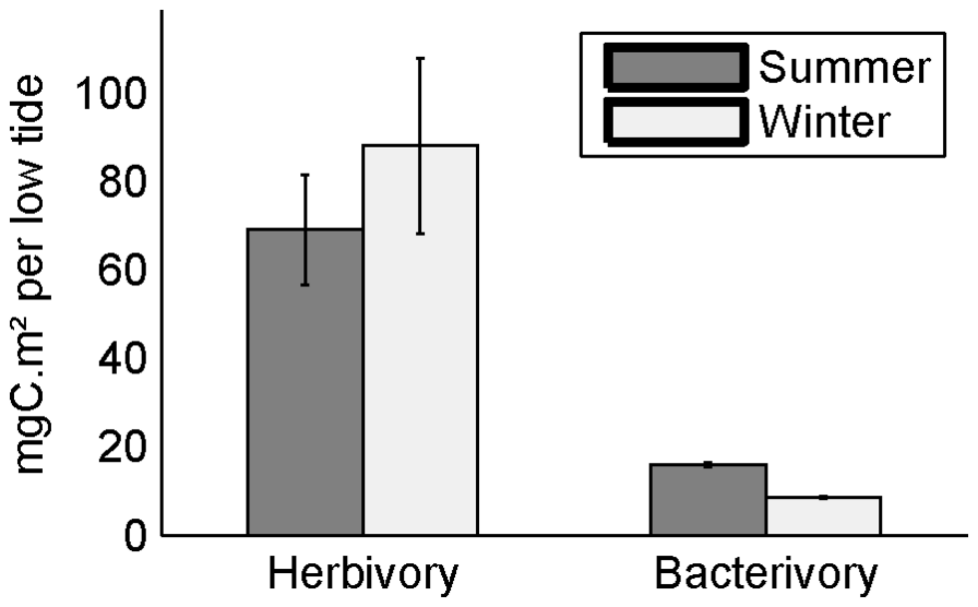
Herbivory and bacterivory in the summer and winter food webs. The herbivory and bacterivory correspond to the mean value of herbivory and bacterivory estimated for the 500,000 solutions proposed by the MCMC-LIM method. The error bars represent the standard deviation of values.

Herbivory appeared more important in winter than in summer, whereas bacterivory increased in the summer (Figure 2). In summer, bacterivory represented a higher proportion of the consumption by benthic organisms, with the ratio bacterivory/herbivory being 23% in summer. Bacterivory decreased in winter, to represent only 9% of herbivory.

**Figure 3:**
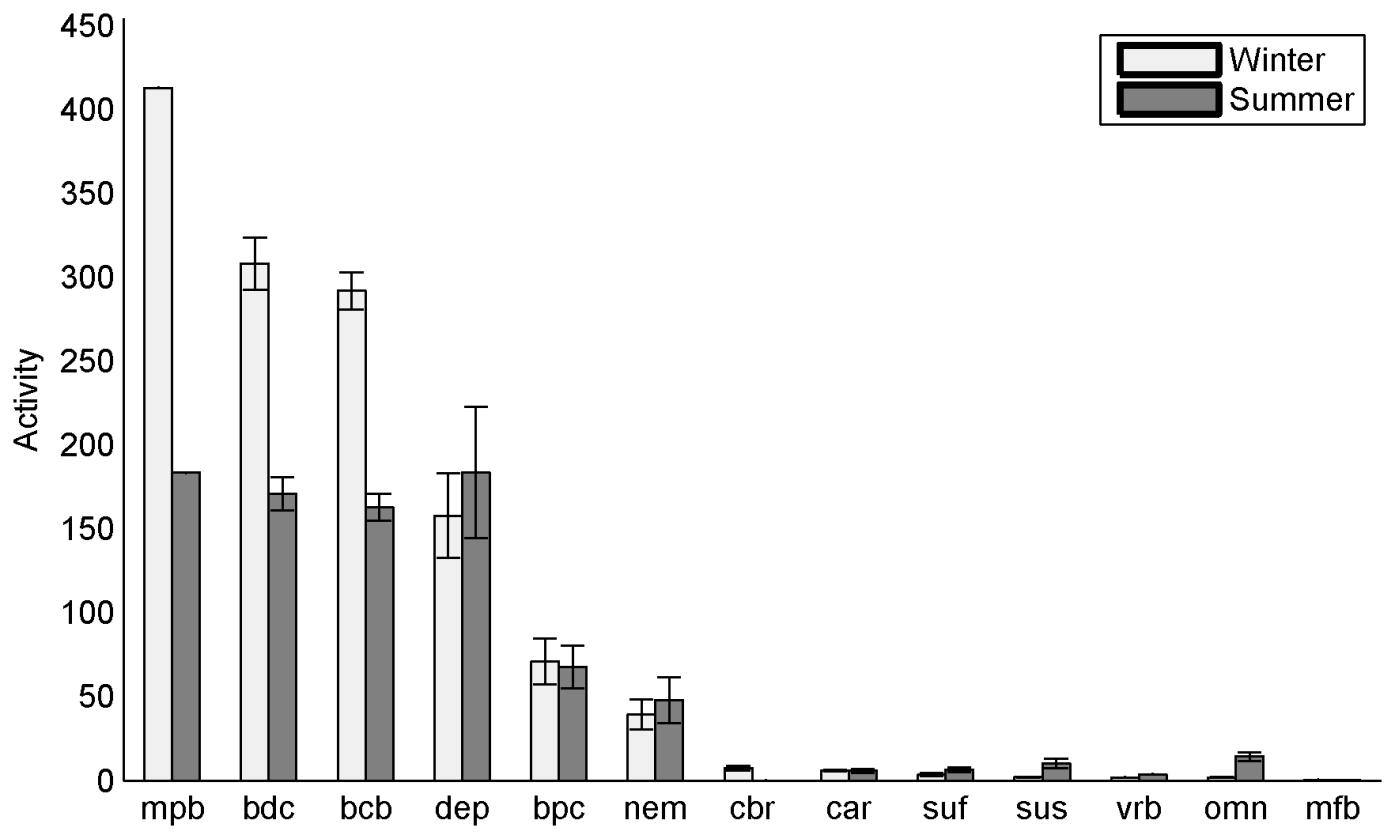
Compartment activities in mgC.m^-2^.LT^-1^ for the two seasons. The error bars represent the standard deviation on the 500,000 simulations. See Table 1 for compartment abbreviations.

The compartment throughputs (sum of inputs) of the various compartments, which quantifies their activity (Figure 3) followed different tendencies. The order of the compartment activities changed according to the season considered. The activity of the biofilm (mpb, bdc and bcb) was higher in winter, in contrast to the activity of macrofauna that tended to be higher in summer. In winter, microphytobenthos dominated the benthic activity, followed by benthic bacteria and dissolved organic carbon. The activity of deposit-feeders was the next highest, followed by that of particulate carbon and nematodes. In contrast, during summer, the four first compartments (mpb, bdb, bcb and dep) demonstrated a similar quantity of inputs. Shorebird activity was ranked between the activity of deposit-feeders and the activity of carnivorous macrofauna.

### 2: Ecological network analysis

**Figure 4:**
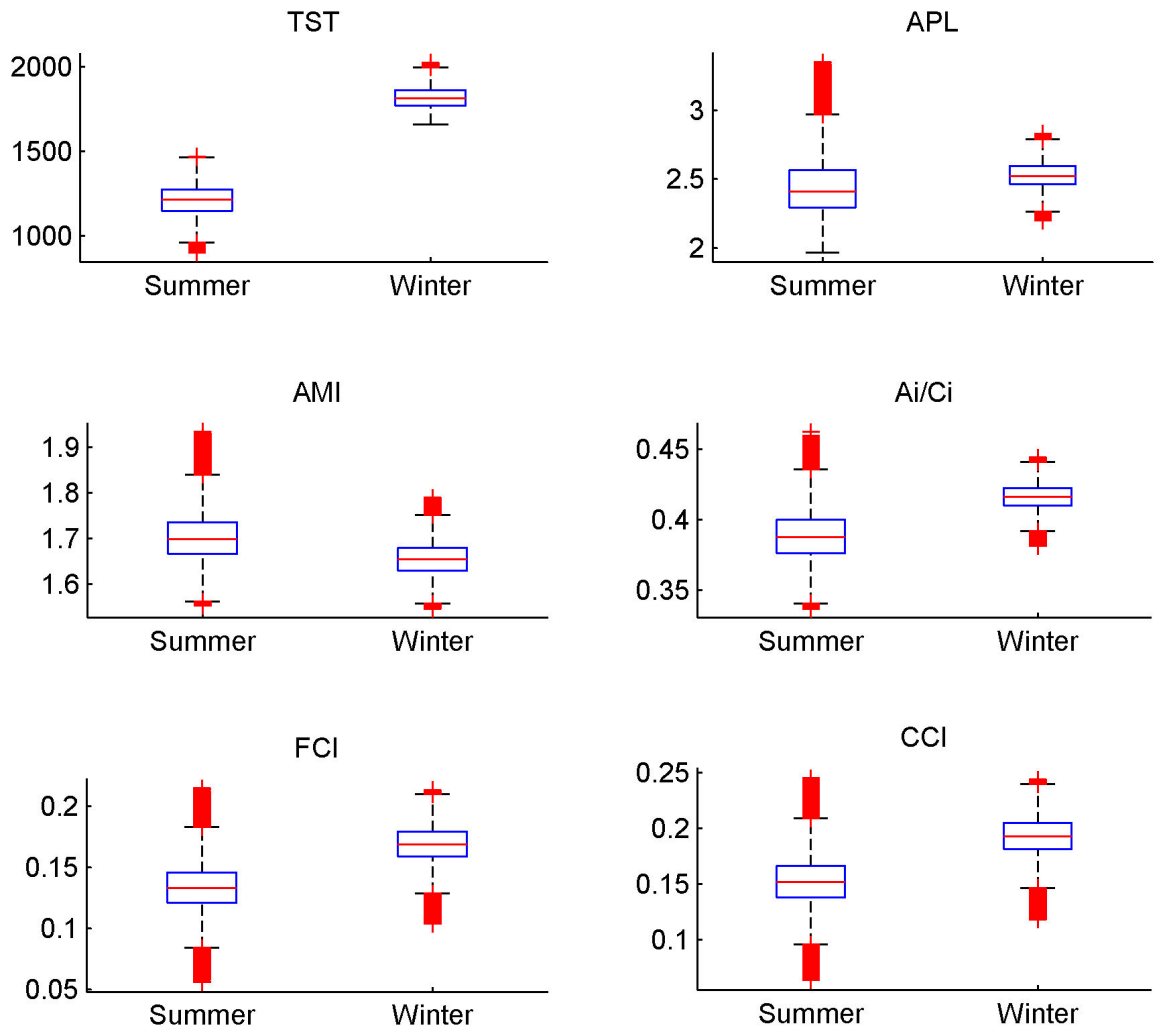
Boxplot representing the ENA indices. The Total System Throughput (TST), the Average path Length (APL), the relative Ascendency (A/DC), the Average Mutual Information (AMI), the internal relative Ascendency (A_i_/C_i_) ,the Finn Cycling Index (FCI). These indices were calculated with the 500 000 solutions of the MCMC- LIM implementation. Red crosses correspond to outliers. Medians of all these indices were significantly different for the two seasons (Wilcoxon test, H_0_ was rejected, p-value < 0.01).

For all the ENA indices calculated for the 500,000 solutions of the MCMC-LIM, values of indices significantly differed between the two seasons (Figure 4). A higher TST was observed in winter, which suggests a higher activity of the whole system. Thus, more carbon and energy flowed through the food web in the winter. A higher quantity of carbon involved in the cycling was observed in winter (i.e. high values of the FCI). The high relative ascendency (A/dC) that ranged from 0.6 to 0.7, independent of the season, suggests a well-organised ecosystem and this organisation tended to be higher in winter. This Relative Ascendency value showed that the organised part of the system was more important than the inefficient part of the network.

For the AMI, APL and A_i_/DC_i_ the trend was less obvious (Figure 4). The ranges of summer values encompassed those in winter, but the Wilcoxon test remained significant. The specialisation of pathways, measured by the AMI, only slightly changed according to the season. The internal organisation that corresponds to the A_i_/DC_i_ value, remained very similar for the two seasons. This indicates that when the exogenous exchanges are excluded, the network showed a similar organisation. The APL defines the mean number of compartments that an atom of carbon passes through before leaving the food web. Again, the difference was small, which means that an atom of carbon passes through a similar number of compartments, irrespective of season.

Complementary indices calculated with the mean value of each flow (Table 6) confirmed the previous trends. This second set of indices was estimated from the EcoNetwrk and as a consequence, no uncertainties were estimated. More numerous flows were involved in cycling in winter, with 48, compared to 28 in summer, which confirms the higher cycling in winter. In spite of its higher activity, a lower mean trophic efficiency was observed in the winter food web. The fraction of inefficient network associated with the relative redundancy was similar in the two food webs and thus confirmed a similar specialisation of trophic pathways. The inefficiency of the network was also measured using overheads: the loss of efficiency due to the imports of carbon into the ecosystem (i.e. overheads on imports), the loss of carbon by dissipation (i.e. dissipative overheads) and the loss of efficiency by export of carbon outside the ecosystem (i.e. overheads on exports). The loss of efficiency due to imports appeared to be lower in winter, whereas the inefficiency due to the exports tended to be higher. The loss of carbon by dissipation was similar for the two seasons.

**Table 6:** Parameters of trophic network in winter and summer.

**Attributes**	**Winter**	**Summer**
Mean trophic efficiency (%)	5.8	6.54
Number of cycles	48	28
Relative redundancy R/DC (%)	23.51	24.08
Overheads on imports (%)	4.97	6.08
Overheads on export (%)	13.02	11.37
Dissipative overheads (%)	10.99	10.82

These indices were calculated from the mean of the values estimated by the MCMCIA method.

**Figure 5:**
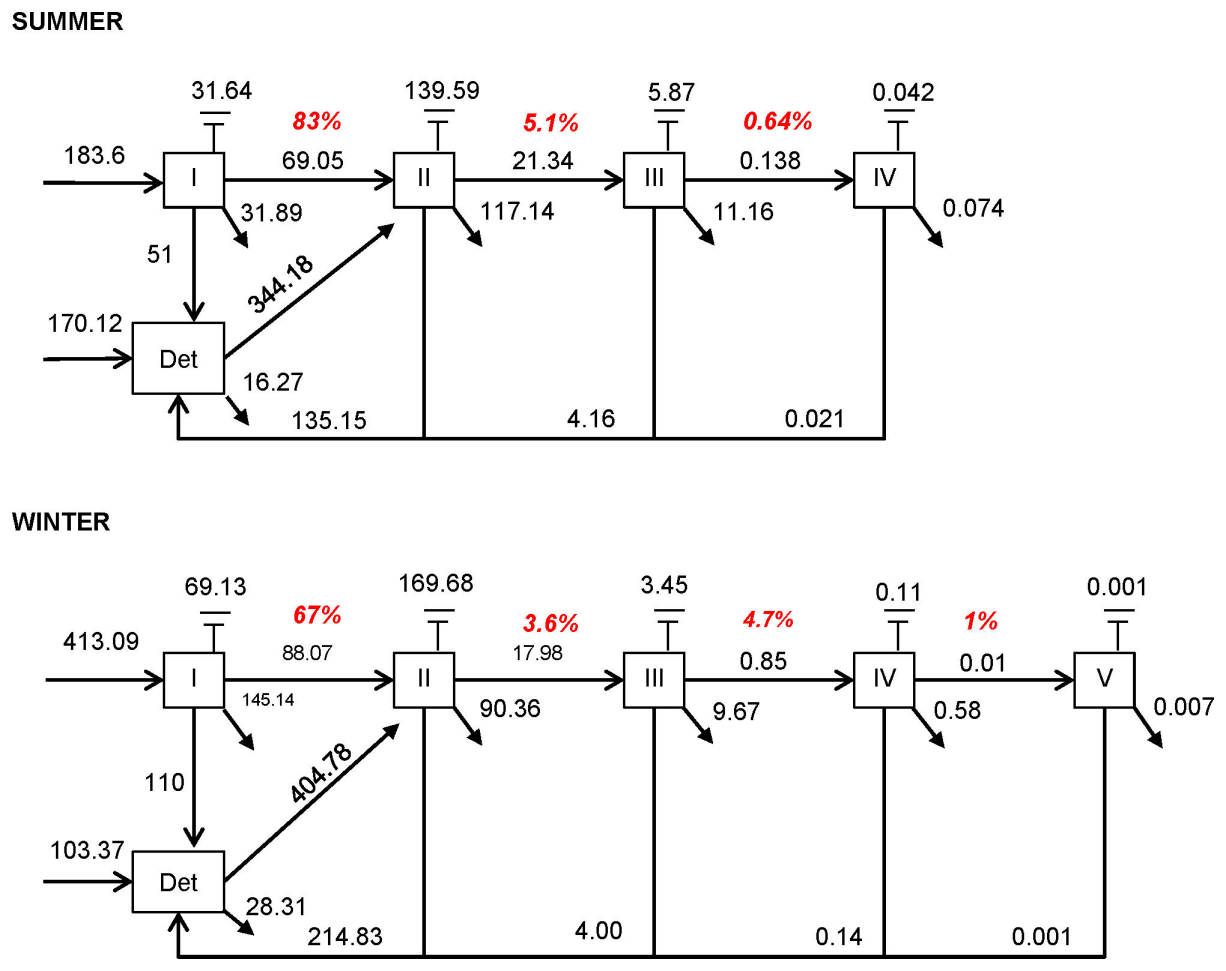
Lindeman Spine for the two seasons. The correspond to the exchanges between trophic levels, the show the export of carbon and symbolize the loss of carbon by respiration. Numbers in red are the transfer efficiencies between each level. Imports to macrofauna were assimilated to imports to the non-living compartment.

The Lindeman spine, which represents the complex network in the trophic chain, comprises four levels in summer and five levels in winter (Figure 5). Level I contained the primary producers, which are the microphytobenthos in this model. The compartment det (detritus) regrouped all non-living compartments, which included dissolved and particulate carbon. Level II grouped herbivores (i.e. a part of the meiofauna, and macrofauna except carnivorous) and bacteria. Levels III and IV contained the macrofauna and the remaining bacterivorous species in level III and carnivorous and omnivorous species in levels III and IV. The shorebirds were in levels III, IV and V, and were the only group in level V.

The inputs to level I were higher in winter, whereas the inputs to the det compartment were higher in summer (Figure 5). The exports from these levels (I and det) were higher in winter. A higher quantity of carbon passed from level I to level II in winter, but this transfer was less efficient than in summer. Detritivory, linking the Det and level II was more important in winter. The respiration of the second level was higher in winter, in contrast to the export, which was smaller in winter. The return to det was higher in winter. Trophic efficiencies between lower trophic levels (i.e. between I and II and II and III), were higher in summer. The winter food web was characterised by a contrasting pattern, with low trophic efficiencies at lower trophic levels and more efficient transfers in the upper part of the chain (between levels III, IV and V).

## Discussion

### 1: Compartment activities

Differences in ecosystem function between winter and the summer have already been observed in the Brouage mudflat [9,10]. In previous works, the summer food web appeared to be the most productive season [10], whereas in the present models, winter was the most productive. This apparent contradiction might be explained by the consideration of a high primary production in summer in the previous models of Degré et al. [10] and Leguerrier et al. [9]. In these models, the ‘summer’ period corresponded to seven months from March to October with a strong heterogeneity of primary production: some months like April with a high productivity [53] and others (June, July) of lower productivity. The integration of this heterogeneity led to a mean production higher than the one considered in our model which is restricted to ‘true’ summer (July). On the Brouage mudflat, July is characterised by strong lights and temperatures that can be harmful for photosynthesis and that can inhibit the productivity of microphytobenthos [53,54,55]. Additionally, high grazing in summer generates a significant depletion of the microphytobenthic biomass [7,56]. Hence, because we considered truly characteristic periods (July and February), the seasonal differences in the microphytobenthic production were different from previous studies [9,10].

The biofilm activity (microphytobenthos, benthic bacteria and dissolved organic carbon i.e. EPS) dominated the benthic activity. This major finding was in agreement with a previous Brouage model developed on an annual basis [31]. The dominance of biofilm activity was true for both seasons and was more pronounced in winter. However, the ranking of compartment activity inside the biofilm differed from before [31]. In the present study, microphytobenthos showed the higher activity, whereas benthic bacteria and DOC dominated in the model from Leguerrier et al. [31]. Because substantial knowledge was since gained on the seasonal variation of bacterial production [17] and on the bacterivory exerted by nematodes, meiofauna and *H. ulvae* [57,58,59], refined estimates of bacterial processes were used here. Shorebird activity remained low in the three models (annual, winter, and our models), and corresponded to 2.2%, 1.4%, 1.7%, respectively, of the primary production [10,31]. In our model, the activity of shorebirds represented a similar proportion of the primary production, 1.6%, as that found in the Rømø-Sylt Bight [32].

The ranking of macrofaunal compartments changed in comparison with the annual model [10,31]. In the present models, macrofauna activity was largely dominated by deposit-feeders mainly composed of *H. ulvae*, the most abundant species of the intermediate mudflat [60] (the sampling station in this study). The previous annual model additionally covered the lower and upper parts of the mudflat, where some species of bivalves such as *Scrobicularia plana* and *Cerastoderma edule* are also abundant [61]. In summer, deposit feeder activity was relatively high in comparison to that of the microphytobenthos being similar to the total primary production. This high activity was due to the high value of carbon imports of 142 mgC m^-2^ per low tide to this compartment. The primary production could not sustain the carbon production of deposit-feeders from the diurnal low tide suggesting that deposit-feeders rely on other resources at high tide. Benthic bacteria can be considered as an alternative resource [59] and detritivory is also a plausible hypothesis: during high tide, detritus can be imported by oceanic waters and/or by two rivers (i.e. La Charente and La Seudre) as shown before [31]. The detrital carbon can be ingested at high tide and imported to the low tide food web. However, the import to deposit feeders (142 mgC m^-2^ per low tide) might be overestimated because the mean was about three times the minimum requirement of 42 mgC m^-2^ LT^-1^. High mean was mostly driven by the high maximum value integrated as an inequality in the model (210 mgC m^-2^ per low tide). The maximum value corresponded to the production estimated from the P/B ratio ranging between 0.01 and 0.05 in the literature [44]. The P/B ratio of 0.05 was an extreme value and might thus overestimated the actual daily production of the deposit-feeders.

### 2: Model choices for birds

The constraints of shorebird processes were based on a range of shorebird densities and on the basal metabolic rate of each species. This choice was made because in winter, shorebird consumption only balanced energy expenditure [45,46]. Moreover, for shorebirds that feed on the intertidal mudflat, distances between the roosts (i.e. rest areas) and the feeding areas are short and limit the energy losses [62]. The P/B ratio estimated in this study was similar to ECOPATH models [63,64]. A model applied to the Bay of Mont Saint-Michel on the Channel coast of France showed a higher P/B ratio for shorebirds, of 0.4 [65]. In studies on seabirds, the lower P/B was equal to 0.09 [63,64] or 0.10 [66]. The ratio of production/consumption (P/C) is extremely variable and can differ by an order of magnitude and the ratio greatly depends on the losses by egestion and respiration. An assimilation efficiency of 80% is currently used [42,67]; in this study, the assimilation efficiency only exceeded this value by 3% (the absolute value of egestion was 1.2 mgC m^-2^ per low tide, compared to 1.5 mgC m^-2^ per low tide with an assimilation efficiency of 80%). Losses of carbon due to respiration vary with bird activity. For seabirds [63,64,66], losses by respiration are important due to the flights during predation activity (i.e. the P/C ratio is low in these models). In this study, the loss of carbon by respiration was chosen to be equal to the minimum metabolic rate. This might constitute a slight underestimation of the respiration of waders and thus, their production might have been greater.

To determine a maximum and minimum consumption by shorebirds, maximum and minimum densities on the mudflat were considered. These two extremes were based on the assumption of a homogenous repartition either on the whole mudflat for the minimum density, or only on the area of the mudflat which is emerged per hour for the maximum density. Nevertheless, the distribution of shorebirds species is dependent on the prey-specific distribution on the mudflat and/or on their foraging abilities [61]. For instance, *Scrobicularia plana* is restricted only to the upper part of the mudflat, whereas *Cerastoderma edule* is located in the lower part while the prey *H. ulvae* colonises the whole area [61]. Because the intra-specific competition and the density of their prey control the spatial repartition of shorebirds on the mudflat [e.g. 68,69], it is difficult to predict precisely the shorebird densities that feed within a given area, and thus to estimate the actual predation pressure per unit area. Due to our current knowledge of the spatial distribution of shorebirds on the Brouage mudflat, there was no alternative than setting two extreme densities to define the range in which the shorebird consumption behaviour might be estimated as a whole.

### 3: Consumption by birds

Considering a whole mean foraging time of 11.3 h, the estimated daily consumption was 20 mgC m^-2^ d^-1^ (or about 34.5 mgAFDW m^-2^ d^-1^). In comparison with the previous winter model of Brouage [10], bird consumption was higher thanks to our better knowledge of bird diets [5,6,47,48]. The estimated consumption in this study is close to that of shorebirds observed in the Wadden Sea, the Rømø-Sylt Bight [42] and the Somme Bay [70]. Moreover, the obtained consumption corresponded to a density of birds on the Brouage mudflat close to the highest determined density, which was 2.05 × 10^-4^ individuals per m^2^ (or 54 mgC m^-2^). This biomass was in the same order of magnitude as that in the Rømø-Sylt Bight for the same assemblage of species [32].

### 4: Food-web function

Whole ecosystem activity, measured by the TST, was significantly higher in winter than in summer. This can be explained by a higher gross primary production in winter (413.09 mgC m^-2^ per low tide) compared to summer (183.6 mgC m^-2^). In summer, the strong light and the temperature conditions generated photoinhibition of the photosynthesis of microphytobenthos [53,71] and lead to a decrease in gross primary production. Additionally, a strong decrease in microphytobenthos biomass was observed [7] due to intense grazing by benthic invertebrates. The high winter TST value was linked to bacterial production which was higher in winter (169.3 mgC m^-2^ per low tide) compared to summer (93.9 mgC m^-2^ LT^-1^). Considering the field bacterial P/B ratio, a value of 0.92 was found during summer in the Brouage mudflat and 0.77 in winter [17]. In parallel, the bacterial biomass doubled in winter and could thus sustain the high production.

The bacterivory and the herbivory of deposit feeders, meiofauna and nematodes changed according to the season. In winter, 84.8% of the carbon ingested by the three compartments came from the microphytobenthos (herbivory); in summer, the proportion decreased to 75%. Bacterivory followed the opposite tendency and was higher in summer. Even with the higher bacterial production in winter, the bacterial carbon was less ingested by primary consumers which preferentially feed on microphytobenthos, regardless of the bacterial production [57,58,59], except when microphytobenthos biomass drastically decreases [17]. This is what happened in summer because the gross primary production was low, as was the carbon available to upper trophic levels.

The trophic pathway lengths were similar for the two seasons (i.e. high APL values). For the winter food web, the shorebird compartment was added. Unexpectedly, an atom of carbon passed through almost the same number of compartments before leaving the system. The path length value is linked to the degree of cycling within the ecosystem [72]. The number of cycles was almost doubled in winter and the proportion of cycles behaved similarly (i.e. a higher FCI value). The stronger cycling can be explained by the increased detritivory behaviour (404.78 mgC m^-2^ per low tide in winter compared with 343.86 mgC m^-2^ per low tide in summer), in particular for bacteria. The bacterial biomass and production were higher in winter, with dissolved organic carbon representing 72% of the whole detritivory that was taken in larger quantity. The stronger carbon cycling lead to a higher retention of carbon inside the ecosystem without changing the carbon path compared to summer. This can be explained by a higher/faster export of carbon. Indeed, one-third of the primary production and twice more of the bacterial and detritic carbon were exported at high tide. The FCI and the number of cycles previously defined for the same seasons at the Brouage mudflat showed an opposite tendency [9] with higher values in summer. In the annual model [9,10], bacteria and detritus (dissolved and particulate carbon) were combined which infers strong implications for the ENA indices [73,74] explaining the difference with our model.

In summer, the higher sum of all imports to macrofauna (168.6 mgC m^-2^ per low tide and 103 mgC m^-2^ per low tide, respectively) suggested that macrofauna was more dependent on imports from other periods of the day than in winter. This finding was confirmed by the relative value of overheads on imports which was higher in summer (6.14%) than in winter (4.96%). The total export of carbon by macrofauna to the high tide was higher in winter as likely explained by a higher summer loss of carbon and respiration by macrofauna. For instance, summer is the recruitment for the abundant *H. ulvae* [75] so that the individuals are smaller and their body mass lower. From the metabolic point of view, organisms with a low body mass have a higher metabolism per unit of biomass and the metabolism increases with the temperature [76] well supporting why more carbon was lost by macrofauna respiration in summer.

The internal organisation and specialisation of the two food webs were similar. This is supported by the similar relative redundancy for the two seasons. The ranges of internal Relative Ascendency, of the specialisation (i.e. AMI) and of the relative redundancy observed in the literature [e.g. 15,77,78,79], include the values for the Brouage mudflat. In winter, a higher specialisation of the trophic pathways was observed for high trophic levels because shorebirds preferentially fed on deposit feeders, and about one quarter of the production of carnivorous, omnivorous, suspension feeders and facultative suspension feeders was ingested by birds. The internal relative ascendency only considers the internal flows of the network [80], i.e., trophic interaction between species. The similar values of this index for the two seasons illustrate a similar organisation of the interactions between species irrespective of the season. However, a higher relative ascendency, which measures the organised and efficient part of the system [80], in winter suggests a higher level of organisation. This index considers all flows of the ecosystem, including internal flows and flows through the boundaries of the ecosystem. Nevertheless, the strong difference between the A/dC ratio and A_i_/DC_i_ ratio suggests a strong dependency of the system on exchanges through the boundaries of the ecosystem [81]. Because this difference is higher in winter, it means a higher dependency of the winter food web to external connections explaining the higher value of relative ascendency, whereas the internal relative ascendency for the two seasons remained similar. Consequently, in winter, a large proportion of the net primary production was exported to the high tide (45%) and a lower proportion was consumed by the main grazers (only 25%). Nevertheless, a higher proportion of the secondary production was consumed due to the high shorebird density. In summer, a large proportion of the net primary production was integrated into higher trophic levels through consumption by nematodes and deposit feeders. However, a low proportion of the secondary production was ingested by secondary consumers. Noteworthy, these two different network patterns still lead to a similar internal organisation.

### 5: The Lindeman spine

A transfer efficiency between the level I+det and level II as high as estimated here (67% in winter and 83% in summer) is not common: the trophic efficiency in the literature tends to be lower than 50%, with a few exceptions [REF]. The transfer efficiency of the Brouage mudflat in summer was close to that found in the Baltic sea [15] and in the mud and muddy-sand flat habitats in the Rømø-Sylt Bight [77]. The high transfer efficiency of the Brouage mudflat was linked to the temporal scale. Indeed, the literature food webs consider a mean per day, whereas in this study, only low tide was integrated. Some transfer of carbon, which derived from the previous low and high tides, also appeared for macrofauna on small time-scale. These imports were assimilated as an import to the det compartment in the Lindeman spine. Thus, a part of det was, by default, transferred with an efficiency of 100% to the higher trophic levels which explains the strong transfer efficiency to level II.

The summer food web was characterised by low primary and bacterial productions. As explained above, the dominant macrofauna *H. ulvae* has a low mean body mass in summer which means that a higher quantity of carbon is necessary to satisfy its metabolic requirements for a given biomass. In winter, an increase in the transfer efficiency between the compartments III, IV, and V was observed, corresponding to an increase in shorebird carnivory. The mean transfer efficiency tended to be higher in summer, nevertheless, the two mean transfer efficiencies were close to those of the mud flat in the Rømø-Sylt Bight [77]. The winter food web was thus highly productive: it exported excess microphytobenthic carbon during the resuspension of the biofilm at high tide (wave and wind-induced currents). The food webs for the two seasons thus showed drastically opposite trends: the winter food web at low tide exported excess carbon, whereas the summer food web led to higher imports. The same conclusions were reached from the previous food web model [10].

### 6: Synthesis

**Figure 6:**
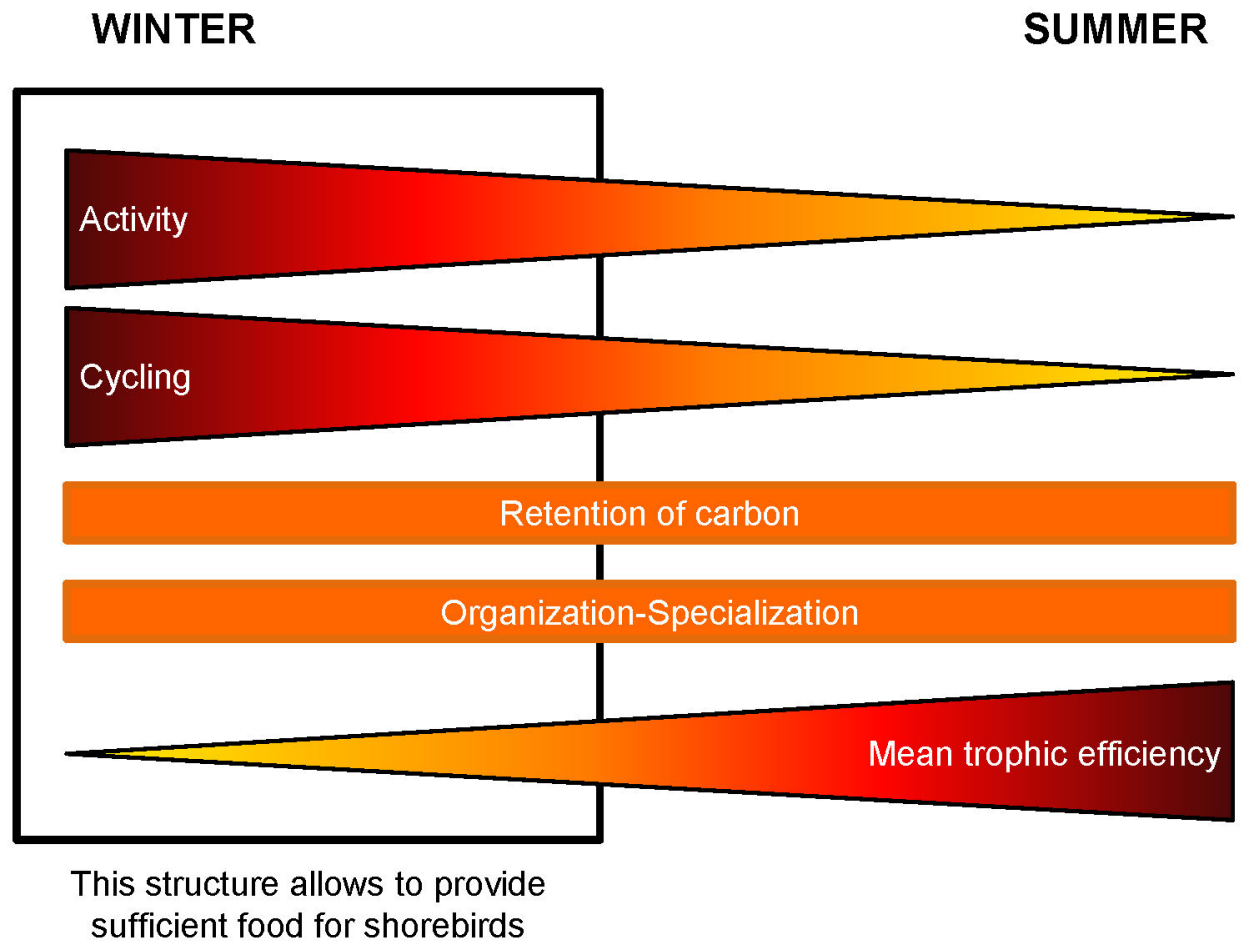
Summary of the observations made on the winter and summer food webs.

Although the summer and winter food webs of the Brouage mudflat showed some similarities, the winter food web possessed specific characteristics that allow the sustainability of the migratory shorebird nutritional needs (Figure 6). First, the internal organisation and specialisation of the trophic pathways were similar for the two seasons. Although the efficient part of the two networks represented the same percentage of the whole activity of the system, it did not include the same levels of the trophic chain. In summer, the strongest efficiency was found for the low trophic levels, whereas in winter, it was found for the higher trophic levels due to the shorebird nutritional requirements. However, the winter food web was characterised by a strong activity of the whole system which was supported by high primary and bacterial productions. In winter, cycling was also stronger due to higher bacterial activity and higher detritivory. Nevertheless, the strong carbon export from microphytobenthos, bacteria and the non-living compartment, led to a similar retention of carbon for both seasons. In spite of the addition of one compartment (i.e. shorebirds) and a stronger cycling in winter, an atom of carbon passed through almost the same number of compartments as in summer. The decrease in primary production observed in summer and probably due to photo-/thermo-inhibition, was compensated for by a higher bacterivory by the meio- and macro-benthos. The strong integration of the carbon from the primary and the bacterial productions to the higher trophic levels led to a stronger mean transfer efficiency. Consequently, the Brouage mudflat showed a similar organisation and specialisation of flows for the two seasons. The efficient part was almost in equilibrium with the inefficient part of the system. In winter, the low carbon integration resulting from primary production was counterbalanced by stronger cycling. Hence, the carbon retention in the food web did not change between winter and summer. The switch to both higher production and cycling, combined with a constant organisation and specialisation of the system, are the key features that support the nutritional needs of migratory shorebirds in winter in the Brouage mudflat.

## Supporting Information

Table S1 **Flow values in the winter and summer models, expressed in mgC.m^-2^ per low tide.** Values in bold correspond to flows estimated *in* *situ*. Values of flows were estimated by the average of 500,000 solutions obtained by the MCMC-LIM implementation. Values expressed in average +/- standard deviation.
(DOCX)
