# Supplementary material for: Key Features of Intertidal Food Webs That Support Migratory Shorebirds
Source: PLoS One. 2013 Oct 25;8(10):e76739. doi: 10.1371/journal.pone.0076739 (PMC3808337; doi:10.1371/journal.pone.0076739)
Supplement: Table S1 — Flow values in the winter and summer models, expressed in mgC.m-2 per low tide. Values in bold correspond to flows estimated in situ. Values of flows were estimated by the average of 500,000 solutions obtained by the MCMC-LIM implementation. Values expressed in average +/- standard deviation. (DOCX) [file pone.0076739.s001.docx]

**Table S1.**

| **Flows** | **Winter** | | | **Summer** | | |
| --- | --- | --- | --- | --- | --- | --- |
| Gross primary production of microphytobenthos | **413.1** |  |  | **183.6** |  |  |
| Consumption of meiofauna | 0.3 | **±** | 0.0 | 0.2 | **±** | 0.0 |
| Consumption of nematodes | 39.3 | **±** | 8.9 | 48.0 | **±** | 13.6 |
| Consumption by carnivorous species | 2.6 | **±** | 0.3 | 2.1 | **±** | 0.3 |
| Consumption by deposit-feeders | 63.5 | **±** | 17.7 | 41.4 | **±** | 6.9 |
| Consumption by omnivorous species | 0.8 | **±** | 0.1 | 5.0 | **±** | 0.7 |
| Consumption by facultative suspension-feeders | 0.8 | **±** | 0.1 | 1.4 | **±** | 0.6 |
| Consumption by carnivorous birds | 7.4 | **±** | 1.5 | - |  |  |
| Doc uptake by benthic bacteria | 291.9 | **±** | 11.3 | 162.9 | **±** | 8.1 |
| Viral lysis | **1.8** |  |  | **3.6** |  |  |
| Exudation of doc by microphytobenthos | **110.8** | **±** |  | **51.0** |  |  |
| Exudation of doc by benthic bacteria | 147.1 | **±** | 12.0 | 69.7 | **±** | 7.4 |
| Exudation of doc by benthic viruses | 0.9 | **±** | 0.5 | 1.9 | **±** | 1.0 |
| Egestion meiofauna | 0.1 | **±** | 0.0 | 0.1 | **±** | 0.0 |
| Egestion nematodes | 36.4 | **±** | 8.6 | 44.6 | **±** | 12.9 |
| Egestion carnivorous species | 1.2 | **±** | 0.3 | 0.9 | **±** | 0.2 |
| Egestion deposit-feeders | 31.3 | **±** | 10.6 | 19.4 | **±** | 5.2 |
| Egestion omnivorous species | 0.4 | **±** | 0.1 | 2.2 | **±** | 0.5 |
| Egestion facultative suspension-feeders | 0.3 | **±** | 0.1 | 0.6 | **±** | 0.2 |
| Egestion carnivorous birds | 1.2 | **±** | 0.4 | - |  |  |
| Transformation particulate carbon to dissolved carbon | 49.5 | **±** | 15.9 | 48.4 | **±** | 11.0 |
| Respiration microphytobenthos | 69.1 | **±** | 30.0 | 31.6 | **±** | 13.1 |
| Respiration meiofauna | 0.1 | **±** | 0.0 | 0.1 | **±** | 0.0 |
| Respiration nematodes | 1.0 | **±** | 0.1 | 0.7 | **±** | 0.1 |
| Respiration carnivorous species | 1.6 | **±** | 0.2 | 1.8 | **±** | 0.3 |
| Respiration deposit-feeders | 45.5 | **±** | 5.4 | 63.7 | **±** | 16.1 |
| Respiration omnivorous species | 0.5 | **±** | 0.0 | 4.3 | **±** | 0.8 |
| Respiration suspension-feeders | 0.7 | **±** | 0.1 | 3.8 | **±** | 0.9 |
| Respiration facultative suspension-feeders | 0.4 | **±** | 0.1 | 2.2 | **±** | 0.6 |
| Respiration carnivorous birds | 0.9 | **±** | 0.4 | - |  |  |
| Respiration benthic bacteria | 122.6 | **±** | 11.3 | 69.0 | **±** | 8.1 |
| Import to carnivorous species | 3.2 | **±** | 0.5 | 3.7 | **±** | 1.0 |
| Import to deposit-feeders | 94.5 | **±** | 15.9 | 142.2 | **±** | 38.8 |
| Import to omnivorous species | 1.0 | **±** | 0.2 | 9.2 | **±** | 2.6 |
| Import to suspension-feeders | 2.0 | **±** | 0.4 | 10.1 | **±** | 2.9 |
| Import to facultative suspension-feeders | 2.8 | **±** | 0.9 | 4.9 | **±** | 1.4 |
| Export microphytobenthos | 145.1 | **±** | 35.6 | 31.9 | **±** | 16.7 |
| Export carnivorous species | 2.0 | **±** | 0.8 | 3.1 | **±** | 0.8 |
| Export deposit-feeders | 76.0 | **±** | 16.7 | 99.6 | **±** | 27.0 |
| Export omnivorous species | 0.5 | **±** | 0.3 | 7.7 | **±** | 2.1 |
| Export suspension-feeders | 0.5 | **±** | 0.4 | 5.4 | **±** | 2.2 |
| Export facultative suspension-feeders | 1.7 | **±** | 1.0 | 2.6 | **±** | 1.1 |
| Export carnivorous birds | 5.2 | **±** | 1.6 | - |  |  |
| Export benthic bacteria | 13.8 | **±** | 12.0 | 8.3 | **±** | 7.4 |
| Export benthic viruses | 0.9 | **±** | 0.5 | 1.7 | **±** | 1.0 |
| Export dissolved carbon | 16.3 | **±** | 13.9 | 8.1 | **±** | 6.9 |
| Export particulate carbon | 12.0 | **±** | 10.1 | 8.2 | **±** | 7.4 |
